# Competing forces of withdrawal and disease avoidance in the risk networks of people who inject drugs

**DOI:** 10.1371/journal.pone.0235124

**Published:** 2020-06-22

**Authors:** Elspeth Ready, Patrick Habecker, Roberto Abadie, Bilal Khan, Kirk Dombrowski

**Affiliations:** 1 Department of Human Behavior, Ecology and Culture, Max Planck Institute for Evolutionary Anthropology, Leipzig, Germany; 2 Department of Anthropology, University of Florida, Gainesville, Florida, United States of America; 3 Department of Sociology, University of Nebraska-Lincoln, Lincoln, Nebraska, United States of America; 4 VP Research Admin Office, University of Vermont, Burlington, Vermont, United States of America; University of New South Wales, AUSTRALIA

## Abstract

We analyze a network of needle-sharing ties among 117 people who inject drugs (PWID) in rural Puerto Rico, using exponential random graph modeling to examine whether network members engage in partner restriction to lower their risk of contracting HIV or hepatitis C (HCV), or in informed altruism to prevent others from contracting these infections. Although sharing of used syringes is a significant risk factor for transmission of these diseases among PWID, we find limited evidence for partner restriction or informed altruism in the network of reported needle-sharing ties. We find however that sharing of needles is strongly reciprocal, and individuals with higher injection frequency are more likely to have injected with a used needle. Drawing on our ethnographic work, we discuss how the network structures we observe may relate to a decision-making rationale focused on avoiding withdrawal sickness, which leads to risk-taking behaviors in this poor, rural context where economic considerations often lead PWID to cooperate in the acquisition and use of drugs.

## Introduction

Sharing injection equipment with another person when using drugs can be risky but can potentially also have some benefits. Common risks include the possibility of infection with HIV or the hepatitis C virus (HCV). These risks compete with the potential benefits (or avoidance of negative consequences) that may come from *not* purchasing clean works, such as saving money and time, or avoiding potentially incriminating possession of paraphernalia. When people use drugs with others, maintaining good relationships with co-injectors, who are often an important source of assistance, may also factor into the decision to share injection equipment [1–3]. These benefits and risks are reckoned by individuals in view of their past, their present, their envisioned future, and the surrounding social environment. Here, we examine the role of the HIV and HCV infection status in structuring needle-sharing networks among people who inject drugs (PWID) in rural Puerto Rico.

Sharing of used needles in the context of injection drug use is one of the leading transmission pathways for HIV and is similarly an important cause of HCV infections in the United States [4]. Several studies have demonstrated that PWID sometimes consider the risk of infectious disease transmission in their needle-sharing practices. For instance, individuals who are not infected may reduce the number of partners with whom they share equipment, a phenomenon which has been called “partner restriction” [5]. Similarly, HIV-positive individuals may engage in “informed altruism” towards other injectors (i.e., preventing others from using equipment after them) [5]. Serosorting, where PWID use needles with seroconcordant individuals (i.e, others with the same infection status), is another form of protective behavior that has been observed in response to both HIV and HCV [6, 7]. However, treatments such as anti-retroviral therapy (ART) may also have an impact on the risk assessments people make when deciding whether to engage in activities that pose a risk of HIV transmission [8].

The decisions PWID make about needle-sharing are made not only in view of infection risk but also in the context of many other factors and constraints. Additional factors that impact needle-sharing decisions include the biology of addiction and the need to manage symptoms of withdrawal [9–12], mental health issues [13–18], economic constraints [9, 19, 20], social relationships among PWID that are an important source of assistance and emotional support [3, 21–26], the social context of drug use such as shooting galleries [27, 28], and the broader legal/political environment which shapes relationships between PWID and law enforcement, health care service providers, and other institutions [29–31]. Not surprisingly, then, sharing of injection drug equipment is a patterned social process [32]. Many of the factors listed above have important short-term consequences for the well-being of PWID, particularly compared to the consequences of blood-borne infections such as HIV, which can take years to have a significant health impact.

These diverse factors combine to impact the decisions PWID make about acquiring and using needles, decisions that have consequences not only for themselves but for other PWID as well. Individual decisions about sharing needles aggregate to generate the network structure and consequently impact disease transmission. The extent to which diseases such as HIV and HCV can spread through shared needles is highly associated with the structure of needle-sharing networks [33, 34]. For example, dense clustering in needle-sharing networks can accelerate disease transmission [35], and dense cores of high frequency injectors (with a tendency to more risky injection practices) have long been suggested to be an important feature of PWID networks [36, 37]. Understanding risk factors for receptive needle sharing (using a needle after someone else) among PWID, as well as the structure of such networks, are therefore priorities for developing effective HIV and HCV prevention strategies.

However, partly due to the difficulty and cost of collecting detailed sociometric data among PWID, studies analyzing risk factors for needle-sharing have generally taken non-network approaches, using logistic regression to model the probability that an individual engages in receptive needle-sharing at all (e.g., [3, 13, 14, 25, 38]). Yet, as highlighted above, PWID make decisions about risk behaviors in view of individual concerns (such as withdrawal symptoms, economic constraints, and infection risk) within a socially and spatially structured environment, including strong relationships that often involve trust and care [39]. Adopting a more detailed network perspective allows us to go beyond individual-level predictors of risky behavior to examine how properties of relationships among individuals (such as similar personal attributes or reciprocity) shape the network structure. In this article, we aim to investigate the potentially competing roles of disease avoidance behaviors (specifically partner restriction and informed altruism), injection frequency and network effects in shaping needle-sharing behaviors in a sample of PWID in rural Puerto Rico, a region where PWID are generally very poor and drug policies are repressive [40]. This new and uniquely detailed dataset allows us to consider both individual attributes (e.g., injection frequency) and network effects, specifically homophily (the tendency for similar persons to have ties with each other), reciprocity (mutual exchange), and transitivity (the “friend-of-a-friend” property), in producing the network structure.

The “Injection Risk Networks in Rural Puerto Rico” project focuses on the risk factors and injection behaviors of a cohort of PWID in a rural area of the island. Located to the south of San Juan, this broader area includes several small towns, which at the time of the study were within the operations area of a syringe exchange program. This study provides a unique but important perspective on PWID and the transmission of infectious disease, particularly in view of the increasing use of opioids in rural areas throughout the US, and associated increases in HIV and HCV transmission [41–43]. In 2010, the CDC reported that over 20% of new HIV diagnoses in Puerto Rico were the result of injection drug use, whereas only 8% of new HIV cases in the continental US were via injection drug use [44]. Rural injection behaviors in Puerto Rico contribute more to this high incidence level than do urban behaviors [45–47].

We focus our analysis on individual and social factors that might increase a person’s risk of using a needle after someone else. We consider the correlation of network ties with injection frequency (as a determinant of an individual’s need for needles), several potential sources of homophily (age, gender, and location), as well as reciprocity and transitivity. Previous ethnographic research with urban PWID has argued for a “moral economy of sharing” among PWID, as a result of financial constraints and the need to manage withdrawal sickness (e.g, [1]), which lead to pooling of resources and sharing of equipment—sometimes with trusted “running partners,” and sometimes opportunistically in places where PWID congregate. Consequently, we expect reciprocity and transitivity to be important features of the networks. To examine the impact of infectious disease on the network structure, we examine two hypotheses for how HIV or HCV infection might impact needle-sharing behaviors. For both HIV and HCV, we first examine whether individuals with negative self-reported infection status had a lower overall propensity to use needles after others (the partner restriction hypothesis). Second, we examine whether individuals with positive self-reported infection status were less likely to have others to use needles after them (the informed altruism hypothesis).

## Methods

### Study background

Phase 1 of this project, conducted between April and June 2015, recruited 315 PWID through respondent-driven sampling. Participants in Phase 1 completed a survey that closely mirrored the CDC National HIV and Behavioral Surveillance (NHBS) of Injection Drug Users Round 3 questionnaire version 13. The data analyzed here stem from Phase 2 of the project, conducted between December 2015 and January 2017. Phase 2 began with recruitment of Phase 1 participants as key respondents. Most key respondents were selected randomly from among Phase 1 participants, although a small number were recruited to purposefully sample persons with different social or economic roles within the local network (e.g., homeless). Key respondents completed an interview procedure that involved a modified version of the Phase 1 questionnaire, as well as a “Network Supplement Interview” in which they were asked to provide information about people they had used injection drugs with in the past 30 days, up to a maximum of nine partners. The participant was asked to create a nickname for each partner they named, and then was asked whether and how often they used a needle after that partner, and whether and how often that partner used a needle after the respondent. Respondents were also asked about sharing of other injection equipment (e.g., cookers and cottons) with each partner, as well as about other aspects of their relationship, such as how long they had known each other. Both Phase 1 and Phase 2 of the study included rapid HIV and HCV testing for participants who consented to take part in testing in addition to the questionnaires.

In Phase 2, our interview process was repeated for each of the co-use partners named by the key respondents whom we were able to contact. For example, key respondent 1 (KR1) listed five people (A1-A5), and then the research team attempted to recruit each of those five people. If person A1 was interested in the study, they then completed the same interview and network questionnaire as the key respondents. However, we did not interview the next wave of recent co-use nominees (those nominated by A1-A5 who were not already named by a key respondent). After interviewing all of a key respondent’s partners, the interviewers would then progress to the next key respondent and repeat the procedure. Key respondents also brought some additional contacts in for interviews, even if they had not named them as a partner. Criteria for inclusion in the study therefore consisted of (1) being an active injection drug user in the study region, and (2) being a key respondent, or being named by a key respondent as a recent co-use partner and/or placed in contact with the team by a key respondent.

The key respondents’ network interviews occurred concomitantly with ethnographic fieldwork that focused on each key respondent for a period of up to two weeks. During this time, the ethnographers accompanied key respondents as much as possible during daylight hours, while respecting personal boundaries set by each key respondent. The goal of these “focal follows” was to observe injection practices, social contacts in the context of drug acquisition and use, and the strategies key respondents and their contacts used to obtain drugs and/or money for drugs. All fieldwork was conducted by three native Spanish speakers, two of whom live in the region, and all three already had considerable experience working with PWID in the region. During data collection, the team operated a store front that served as a social space for research participants as well as providing opportunities for recruitment.

The co-injection partners named by the key respondents were recruited to the study through a combination of peer-recruitment and ethnographic work. In the course of the focal follows with key respondents described above, many of the people named by participants would interact with the participant and the field staff, and so had a chance to be recruited into Phase 2 of the study. There were also instances where the field staff asked a key respondent to let a partner they listed know that they qualified for the study and where the field team could be reached. Finally, many of the partners listed by key respondents were already known by the field staff from Phase 1 of the project. In these cases, field staff often had ongoing relationships with these participants and would inform them that they qualified for the Phase 2 study and setup a time for an interview if they were interested. All of the eligible persons our team contacted or who were brought in contact with us by other participants agreed to participate in the study (see S1 Appendix). This is not to say that recruitment was easy, however, as many potential participants could not be contacted due to treatment and incarceration, among other factors. All participants provided written consent. Study protocols were approved by the Institutional Review Boards of the University of Nebraska-Lincoln (IRB# 20131113844FB) and the University of Puerto Rico (IRB# A8480115).

### Analysis methods

As with previous studies of PWID networks (e.g., [48]), although our data were collected over a period of several months, we treat the resulting network as if it were synchronous. However, our referral procedure for recruiting participants (described above) meant that closely associated individuals were usually interviewed around the same time, so clusters within the network should generally reflect a relatively synchronous set of affiliations.

We analyze needle-sharing in our study sample using Exponential Random Graph Modelling (ERGM), a method that allows network structures to be modelled in a framework that is similar to logistic regression, where the outcome variable is the probability of an edge between two individuals [49, 50]. ERGMs allow us to model the probability that an individual uses a shared needle using individual-level predictor variables, as well the properties of relationships, such as homophily. By using Markov Chain Monte-Carlo simulation, ERGMs can also estimate higher-level dependencies, including reciprocity and transitivity.

Generally, we focus our modeling procedure on understanding what shapes the in-degree of individuals in the network, meaning the number of other people that an individual used a needle after, as it is using a shared needle, not giving one away, that poses a risk of infection. We begin with a model that includes injection frequency as an individual-level predictor of a PWID’s risk of receptive needle sharing, as well as several potential sources of homophily (age, gender, and location). For each of the homophily terms, we include a degree term to control for the overall propensity of the group to use shared needles, as homophily terms are more easily interpreted in conjunction with these terms. For gender, in view of the fact that over 90% of participants were men, we consider only homophily among women in the models. We model age-based homophily using an “absolute difference” term that examines the effect of the absolute age difference between two individuals on the probability of a tie between them. This approach assumes that individuals with a smaller age difference are more (or less) likely to be connected, but makes no assumption about the directionality of the tie between them (i.e., whether an older person uses a needle first or second). We consider location homophily only for one of the rural counties (*municipios*) included in the study, as a model with terms for all locations would be overfit, and we have a clear reason to expect stronger homophily at this location due to the presence of an active shooting gallery at the time of our study. Finally, our injection frequency variable is an ordinal variable divided into four levels which result from binning together PWID with low injection frequencies because they were less common in the sample (see Results).

For higher-level dependencies, we model reciprocity in the networks using the “mutual” term in ERGM, which estimates the probability of a tie from person *A* to *B* given that a tie from *B* to *A* was already observed. We model transitivity using geometrically-weighted edgewise shared partnerships (GWESP) [51]. This term models whether individuals with common partner(s) are more likely to be connected to each other (leading to triangles), regardless of the tie direction (i.e., whether triads are transitive).

Using the model including reciprocity and transitivity as a baseline, we then run additional models to test the hypotheses outlined in the introduction. First, we examine whether individuals whose self-reports suggest that they are HIV or HCV negative had a lower overall propensity to use needles after others (“partner restriction”). Second, we test the informed altruism hypothesis using an out-degree term to determine whether individuals who report being HIV or HCV positive have fewer partners who use needles after them. Our HIV and HCV data are based on individuals’ self-reports of their previous HIV/HCV test results, as well as their reporting of any treatment for HCV. We use these self-reports rather than our rapid-testing results to examine needle-sharing behavior, as we expect participants’ knowledge of their infection status (rather than a new test result) to have impacted the behavior reported in the survey.

We conducted the analyses in R version 3.6.0 [52] using ergm package version 3.10.4 [53]. ERGMs with dyadic- or higher-level dependency terms (i.e., reciprocity and transitivity) are fit using Markov-Chain Monte-Carlo simulation, as implemented in ergm. These models were run with an MCMC sample size of 400000, a burn-in period of 100000 and an MCMC sampling interval of 100000. For the first model where we consider transitivity, we ran a Curved-Exponential Family model to estimate the decay parameter (*α*) as well as the GWESP coefficient. We then used the same decay parameter (*α* = 0.04) in all subsequent models. Trace and density plots of the MCMC samples, as well as Geweke’s burn-in diagnostic and sample statistic auto-correlations, were examined for all models fit using simulation. For all of the models, we assessed goodness-of-fit using simulations to compare properties of the observed network with networks generated using the estimated model (we examined in-degree, out-degree, edgewise-shared partnerships, and the model statistics). All models converged and none showed signs of degeneracy [54]. We describe our ERGM results below using plots of the model estimates and odds-ratios with 95% confidence intervals, as well as using predicted tie probabilities for illustrative examples. The data and R code used to generate the ERGM results, including model diagnostics and figures, are provided as Supplementary Files.

## Results

### Data description

**Fig 1:**
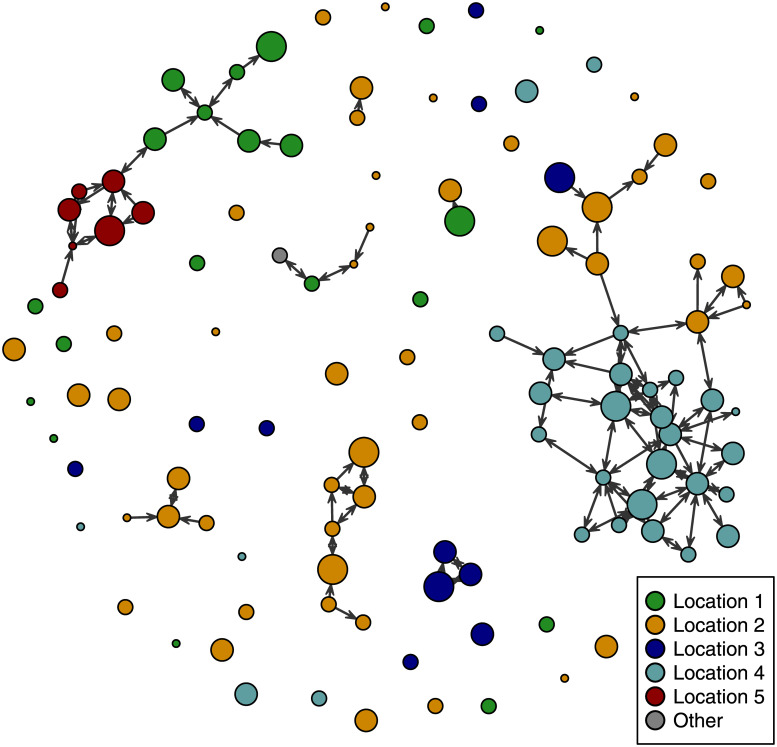
The rural Puerto Rico needle-sharing network. Nodes (individuals) are colored by the residence location of the individual represented. Edges are directed, with arrows pointing to the individual who used a needle after the other person. The size of nodes reflects the frequency with which individuals were using injection drugs, with larger nodes representing network members with a higher injection frequency.

The network we examine here comprises needle-sharing ties among 117 individuals who participated in Phase 2 of the study (106 men and 11 women). The network is directed, with edges going from the first to the second user of the needle according to the reports of respondents. We include all reports of needle-sharing (values of “rarely,” “about half the time,” “most of the time,” and “always”) as an edge in the network, regardless of whether it was a self-reported tie, a tie reported by a partner, or both. We assume that no needle-sharing ties exist where neither of two individuals named each other as a co-injection partner. The resulting network (Fig 1) is composed of 151 ties. 116 (77%) of these ties are part of reciprocal needle-sharing dyads, where both partners use a needle after the other on occasion. Respondents’ in-degree (the number of persons they used a needle after) ranges from zero to nine other persons, with a mean of 1.29. Further details on the sample and the construction of the network can be found in S1 Appendix. Although our study area focused on four *municipios*, each corresponding to an area of roughly 30 to 50 square miles, a small number of participants were resident in other locations, mostly in one other *municipio* (Fig 1).

Among the 117 individuals in the dataset, 18 (15%) reported an injection frequency of less than once a day, 49 (42%) reported injecting between one to three times a day, 38 (32%) between four to seven times a day, and 12 (10%) eight or more times per day. Four percent of respondents (*n* = 5) reported being HIV-positive, and the rapid tests showed that these self-reports were highly accurate (100%, excluding one individual who reported an unknown status but tested negative). 80 respondents (68%) reported a previous positive test for HCV, while 31 respondents (26%) reported a previous negative test for HCV (including four persons who reported being cured of a past infection). Six respondents (5%) reported that they had not previously been tested for HCV. However, 85% of respondents (93/110) who participated in Phase 2 testing for HCV antibodies tested positive (again counting the four reported cured cases as negative tests). Given the limitations of antibody testing, it is not clear which individuals in our sample had active HCV infections. We discuss this issue further in S2 Appendix. Nevertheless, we use the self-report data to analyze needle-sharing because we expect respondents’ beliefs about their infection status to impact their behavior.

76% of the PWID in our sample (89/117) reported having recently used the syringe exchange program that was operating at the time of our study. Almost 50% (58/117) of individuals in the sample reported that concern about contracting HIV was their number one worry, nearly as many as reported all other reasons combined (including becoming homeless, going to jail, affording their fix, overdosing, contracting Hepatitis C, and being able to buy food).

### Homophily, reciprocity, and transitivity

**Fig 2:**
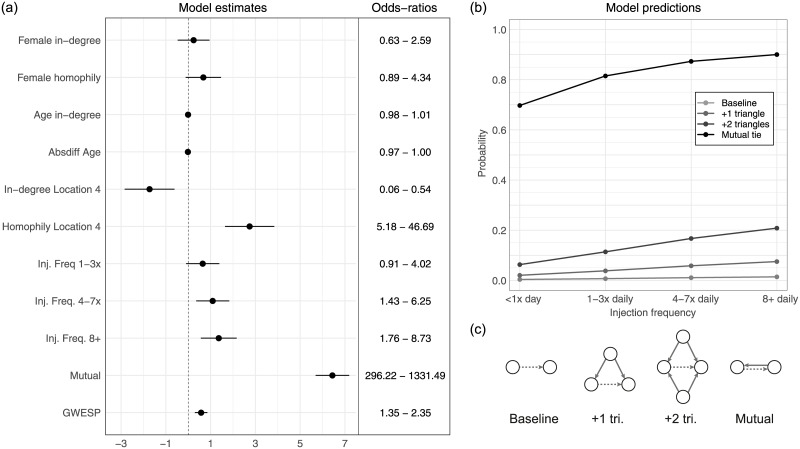
Summary of ERGM model examining gender, age, and location homophily, injection frequency, reciprocity and transitivity (Table 1, Model 3). (a) Estimates and odds-ratios (with 95% confidence intervals) of model coefficients. (b) Prediction of probability of a receptive needle-sharing tie as injection frequency increases, also showing the effects of edgewise shared partnerships and reciprocity, for a 30 year-old male with a same-sex, same-age partner, both in location 4. (c) Illustration of tie types considered in model predictions (dashed arrows). Tie direction for existing edges in the triangles is not important.

Table 1 shows the ERGM results for an initial model considering only homophily terms and our basic “risk” term (injection frequency), followed by models adding terms for reciprocity and transitivity. As expected, we find that higher injection frequency leads to a greater risk that an individual will use a needle after someone else (illustrated in Fig 2 using Model 3), although the differences between groups are small. Age appears to have no (linear) relationship with needle usage, nor do women tend to have a higher overall in-degree. The results suggest a small amount of gender homophily among the women in the network, as well as a slight tendency for the probability of a tie to decrease as the age difference between individuals increases (Model 1); but these associations lose strength when the dependency terms are added. Co-residents of Location 4 are much more likely to have ties with each other than with respondents not resident in the same location.

**Table 1:** ERGM results for models with homophily parameters and injection frequency, reciprocity, and transitivity.

	Model 1			Model 2			Model 3		
	(Base model)			(+ Mutual)			(+ Transitivity)		
	Coef.	S.E.	*p*-value	Coef.	S.E.	*p*-value	Coef.	S.E.	*p*-value
Edges	−5.47	0.62	<0.01	−6.54	0.60	<0.01	−6.44	0.55	<0.01
Node in-factor Female	0.20	0.36	0.58	0.24	0.37	0.51	0.24	0.36	0.50
Node match Female	1.89	0.53	<0.01	0.79	0.45	0.08	0.68	0.40	0.09
Node in-factor Age	−0.01	0.01	0.29	−0.01	0.01	0.34	−0.01	0.01	0.43
Absdiff Age	−0.03	0.01	0.03	−0.02	0.01	0.07	−0.01	0.01	0.08
Node in-factor Location 4	−1.69	0.60	<0.01	−1.83	0.55	<0.01	−1.73	0.57	<0.01
Nodematch Location 4	4.54	0.59	<0.01	3.34	0.53	<0.01	2.74	0.56	<0.01
Node in-factor Injection 1–3×	0.75	0.41	0.07	0.75	0.40	0.06	0.65	0.38	0.09
Node in-factor Injection 4–7×	1.34	0.40	<0.01	1.37	0.39	<0.01	1.09	0.38	<0.01
Node in-factor Injection 8+ ×	1.67	0.44	<0.01	1.71	0.43	<0.01	1.37	0.41	<0.01
Mutual				6.61	0.37	<0.01	6.44	0.38	<0.01
GWESP							0.58	0.14	<0.01
GWESP decay (*α*)							0.04	0.26	0.89
AIC	1347.08			968.22			950.78		
BIC	1422.24			1050.90			1048.48		
Log Likelihood	−663.54			−473.11			−462.39		

Fig 2a illustrates the results for the model including both reciprocity and transitivity. The odds-ratio for reciprocity in the networks is exceptionally high (Fig 2a), which is not surprising given the high number of reciprocated ties in the network (77%). The inclusion of the reciprocity terms leads to a large improvement in model fit. The GWESP term for transitivity is also positive, suggesting that, among individuals who are not isolates, triangles are an important structural feature of the network. Fig 2b illustrates model predictions for the probability of receptive needle-sharing based on the person’s injection frequency, existing shared partners, and whether or not needle-sharing is reciprocated. Fig 2c graphically explains the meaning of the different types of tie predictions considered in Fig 2b. Both increased injection frequency and shared partners increase the probability of a tie between two individuals, but the impact of reciprocity is clearly the most pronounced, with the probability of a tie increasing from less than 1% to nearly 70% for a low frequency user.

### HIV

**Fig 3:**
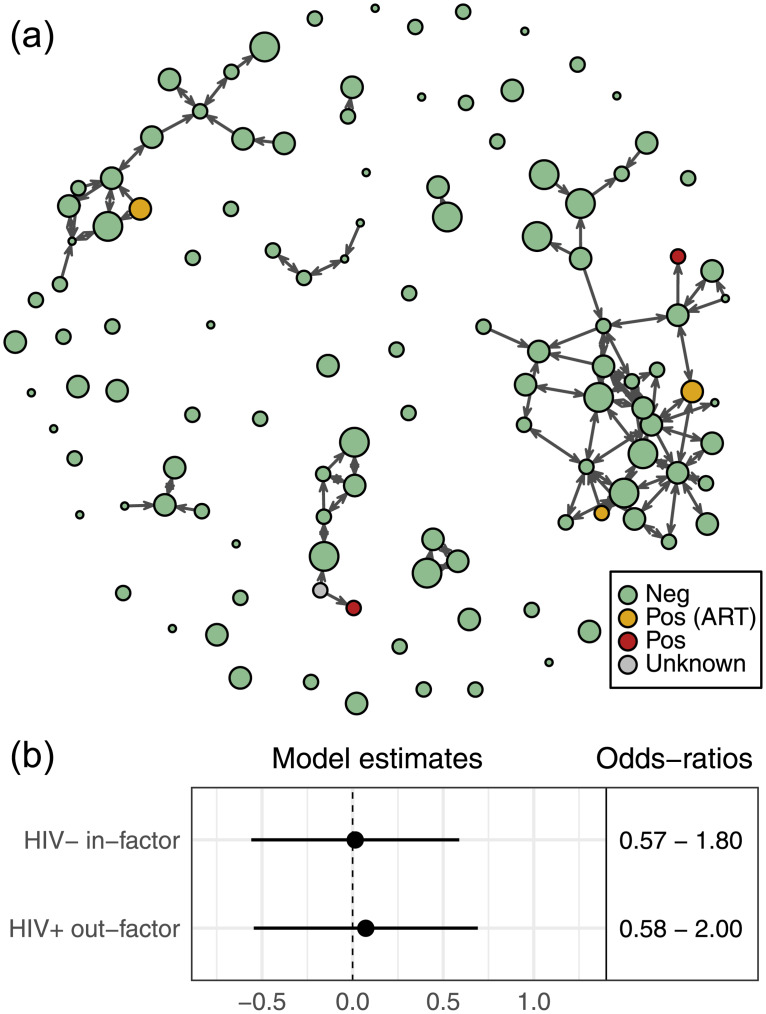
HIV infection and needle-sharing. (a) Needle-sharing network colored by self-reported HIV status. “Pos (ART)” denotes individuals on antiretroviral treatment. The person represented by the grey node did not know their HIV status. (b) Estimates and odds-ratios of model coefficients (with 95% confidence intervals) for the partner restriction and informed altruism terms from the ERGMs considering HIV status.

Fig 3a shows the position of HIV-positive individuals in the network. Visual inspection of this network suggests that needle-sharing with HIV-positive persons by a small number of individuals with high-degree means that a substantial portion of some network components could be at risk of infection through shared needles. For instance, most of the individuals in the component at the top left of Fig 3a are “downstream” from the single currently infected person in the group.

ERGM results examining how self-reported HIV status may impact needle-sharing behaviors are summarized in Fig 3b. The full results for these models are provided in S3 Appendix Table A1. Our first hypothesis, partner restriction, is not supported: HIV-negative individuals do not have a lower probability of using a shared needle than HIV-positive persons (Fig 3b, “HIV− in-factor”). Nor do we find any evidence for informed altruism among HIV-positive persons, as they do not appear to have lower out-degree than HIV-negative persons (Fig 3b, “HIV+ out-factor”). In both cases, the estimated coefficients are close to zero.

We note, however, that all three of the HIV positive individuals who were observed to be a source of needles for others in the sample also reported that they were taking anti-retroviral therapy. The two individuals who were HIV-positive but who reported that they were not on anti-retroviral therapy only had incoming needle-sharing ties (i.e., they used others’ needles, but no one used needles after them). This suggests that if informed altruism to prevent HIV infection is occurring in the network, it may be mediated by antiretroviral treatment, not by HIV status itself. We do not have information on the actual viral loads of any HIV-positive participants, however, and our small sample size of HIV-positive individuals prevents us from drawing firm conclusions.

### HCV

**Fig 4:**
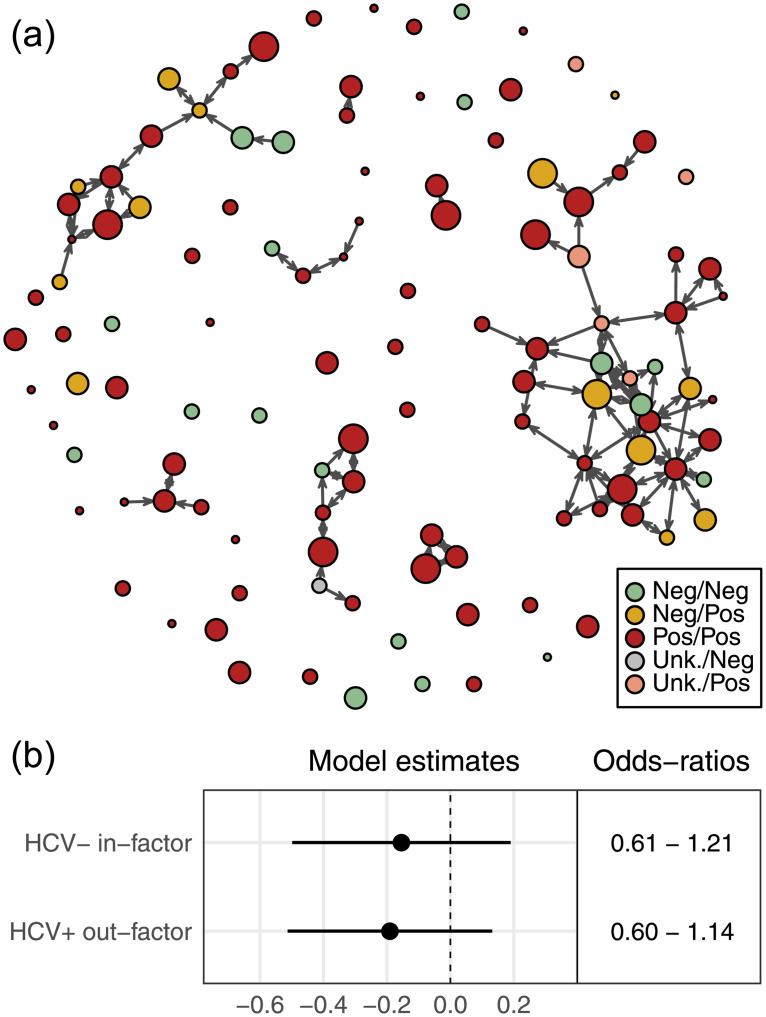
HCV infection and needle-sharing. (a) Needle-sharing network colored by HCV status, shown as the combination of self-report and antibody test results (described in S2 Appendix). (b) Estimates and odds-ratios of model coefficients (with 95% confidence intervals) for the partner restriction and informed altruism terms from the ERGMs considering self-reported HCV status.

Fig 4a shows the networks with individuals colored by their HCV status, while Fig 4b shows the relevant model coefficients for ERGMs examining the partner restriction and informed altruism hypotheses. S3 Appendix Table A2 provides full summaries of these ERGM models.

We find no evidence for partner restriction among HCV negative respondents in the self-report data: participants who reported that they were HCV-negative did not use needles after fewer partners overall compared to HCV-positive individuals. We also find no evidence that self-reported HCV-positive persons have lower out-degree than HCV-negative individuals.

## Discussion

Our analyses suggest that greater injection frequency increases the risk of receptive needle-sharing ties in our sample, and that reciprocity and transitivity are important network features. At the same time, our models provide no evidence for partner restriction or informed altruism in the context of needle sharing. In the following discussion, we draw on our team’s ethnographic experience and fieldnotes to interpret the particular structure of the network and to explain our negative results.

### Needle-sharing in social context

Our results suggest that greater injection frequency increases the risk of receptive needle-sharing ties in our sample. As a PWID’s injection frequency increases, so does their need for drugs and equipment and for money to purchase them. Faced with limited monetary resources, PWID may prioritize purchasing drugs over purchasing clean equipment [9, 19], despite being aware (and afraid) of the risk of HIV infection. In our study, we observed this particularly in participants undergoing the painful effects of heroin withdrawal. Their priority tends to be in securing the resources to “cure” themselves of withdrawal symptoms, and so “completing” the money needed to afford their dose becomes their main concern. Even $1—the price of a new syringe in a shooting gallery or on the street—can set them back in achieving this goal (at the time of our study, a small bag of heroin sold for $6, and cocaine for $5). In this context, borrowing a syringe after someone else has used it is the cheapest and speediest option for a PWID to obtain their “cure.”

Moreover, the need to acquire drugs while having limited money to pay for them also draws PWID into social interactions with others in a way that considerably increases their risk of using shared equipment. In Puerto Rico, drug users often pool their resources with others in a practice called *caballo* (or “horse”) in order to purchase supplies jointly. *Caballo* is practiced in a variety of substance use contexts, for instance for purchasing a joint or even a pitcher of beer. Within our study sample, the ethnographers observed that *caballo* for the purpose of acquiring injection drugs was most often practiced by PWID with a high injection frequency. Such sharing is further encouraged by the fact that most PWID in the area prefer to inject speedball, a combination of heroin and cocaine, which increases the funds required to secure a dose.

Participating in *caballo* can make it easier for PWID to get access to drugs and so facilitates their ability to manage the biology of addiction (i.e., avoiding withdrawal sickness). But in the context of injection drug use, *caballo* carries a host of risks, both social and biological. When pooling drugs in this way, shared cookers are generally used to prepare the solution, which may then be backloaded from another persons’ syringe. In this context, sometimes, although infrequently, needles may be passed from one person to another. The person who contributed the most money to the *caballo* is usually given the privilege of using their own equipment and dividing up the drugs, which allows them to reduce their own risk of infection. This means, however, that most *caballo* participants will eventually use drugs prepared and distributed using someone else’s equipment. *Caballo* therefore may sometimes lead PWID to accept risks that they view as undesirable—such as injecting with a shared needle—in order to ensure that they receive their share. In doing so, they take a small risk with longer-term consequences (of sickness from HIV or HCV) to solve the more proximate problem of obtaining their cure.

Cheating, where participants in a pool steal the money or drugs, or employ tricks to get more than their fair share, is also a common feature of *caballo*. Study participants frequently complained about such cheating. Partly because of the problem of cheating, some PWID in the network choose to rely on a few trusted *caballo* partners. These close relationships may lead partners to be more willing to use a needle after one another. Combined, the rotating order of injection among *caballo* participants and the trusting nature of relationships among “fixed” *caballo* partners likely contribute to the high degree of reciprocity observed in needle-sharing in the network.

In fact, the role of trust in *caballo* may also contribute to the high proportion of isolates in the network. Our ERGM models slightly underestimate the proportion of isolates in the network, and differences in trustworthiness are one possible contributor to this pattern. Some isolates in the needle-sharing network are likely isolates not because they have deliberately adopted protective behaviors, but because they are actually social isolates as well. Such social isolation may result from repeated failures to follow the rules of *caballo*, or from past disagreements that prevent some individuals from being able to secure willing *caballo* partners in the first place.

Triangles are also an important feature of the network. While some clusters of triangles in the network may represent small groups of fixed or recurring *caballo* partners, triadic closure is likely further fostered in the “network core” by two processes: (1) an opportunistic strategy pursued by high injection frequency PWID who engage in *caballo* with temporary partners whenever an opportunity arises, and (2) interactions at a shooting gallery that was a major hub of activity during our fieldwork. Here, large groups of PWID affiliate (and often participate in *caballo* with one other), leading to a dense network centered around a few individuals with high needle-sharing degree.

### Needle sharing and infection risk

Our network analyses do not suggest that HIV-negative individuals in our study sample engage in partner restriction to reduce their risk of contracting HIV, or that HIV-positive individuals engage in informed altruism by preventing others from using needles after them. This may be related to the fact that HIV prevalence is relatively low in this population (4%) compared to other populations of PWID, and so the risk of contracting HIV is relatively low [55]. Yet, both our survey data and our ethnographic experience suggest that PWID in this population are deeply concerned about contracting HIV, which they refer to as *el perro* (the dog) or *la condición* (the condition), and consequently, they avoid sharing needles when possible. Indeed, partner restriction in response to HIV may be occurring in our study population, even if not captured in our analysis. This is because of the possibility that, in effect, all, or nearly all, of the members of the network are reducing their use of shared needles relative to what it would be if HIV infection were not a risk. It seems likely, then, that there is much less needle-sharing than would be the case if HIV were not a risk, but that the situational social and economic considerations discussed above nevertheless lead some PWID to occasionally use shared needles despite the known risks. Our analyses based on HCV infection status also provide negative results; with no evidence of partner restriction among HCV-negative participants or informed altruism among HCV-positive participants.

Most of the PWID in our sample are well aware of the health risks of injecting with a shared needle, and indeed, a large proportion of PWID in the network did not use shared needles at all (or at least did not report that they did). But among those who did, we find no evidence that they selected partners in order to reduce their risk of contracting HIV or HCV, or that PWID with these infections prevented others from using needles after them. In these cases, the situational factors discussed in the previous section are likely more important in shaping the decisions they make about using a needle after someone else.

### Limitations and conclusion

Our study has several limitations. First, our network comprises only a subsample of PWID in the region. Although we started our sampling process for the network interviews with what we believe is a representative subsample of PWID in the region, we cannot demonstrate that the structure of the network we analyze here is representative of the broader network structure. Second, some groups of interest within our sample (e.g., HIV-positive individuals) are small, meaning our data may not provide sufficient information to accurately characterize patterns of behavior among these groups of people. Third, social desirability bias is a potential problem in research that involves stigmatized behaviors [56]. Sharing of needles may therefore be under-reported in the questionnaire, as PWID in the region are aware that syringe sharing is a practice that is discouraged. However, our team had established relationships of trust with local PWID, which allowed the ethnographers to observe a wide variety of social interactions among many PWID, as well as drug use in different locations, from shooting galleries to homes, cars, and street corners. The ethnographers’ observations suggest strong consistency in the behaviors of PWID regarding sharing of drug paraphernalia: sharing of needles is avoided when possible, but economic and other constraints, and the social context of drug use, draw PWID into risk practices. Even if sharing of needles is underreported, we find that the network structure is consistent with our ethnographic observations: many isolates, with dense clusters associated with higher-frequency users and shooting galleries.

Despite these limitations, we feel that our conclusions are likely relevant to other contexts. Though PWID in our study attempt to avoid sharing needles, economic, social, and biological factors nevertheless draw them into risk behaviors, resulting in networks that are not obviously shaped by avoidance of disease transmission. Although some specifics of *caballo* practice in rural Puerto Rico may be unique, sharing of drugs between PWID is a common phenomenon (e.g,. [1, 36]). For PWID in rural Puerto Rico, needle-sharing is shaped by the combination of dependence and poverty that often brings them together through the practice of *caballo*. Sharing injection drugs through *caballo* carries its own risks (e.g., using shared cookers) but can also lead individuals to take additional risks to ensure that they get their share. Sharing drugs and equipment with other PWID is also sometimes associated with longer-term, trusting, reciprocal relationships, which may contribute to the high level of reciprocity observed in the network. The more opportunistic *caballo* strategy of some network members and the social context of shooting galleries likely contribute to the high prevalence of triangles in the high-density components observed in the network. These results speak to the importance of the social and economic context of drug use in shaping risk practices, and underscore the need for prevention, treatment, and harm reduction efforts to consider the social and economic contexts of risk practices and not simply the practice itself. To this end, greater availability of needle exchange programs or safe injection sites could potentially help reduce engagement in some of the riskier practices associated with *caballo*, such as use of shooting galleries and sharing of equipment.

## Supporting information

S1 Appendix Composition of the needle-sharing network. (PDF)

S2 Appendix HIV and HCV testing data. (PDF)

S3 Appendix ERGM summary tables. (PDF)

S1 Data Network data. (CSV)

S2 Data Individual attribute data. (CSV)

S1 Code R code. (R)
