# Supplementary material for: Competing forces of withdrawal and disease avoidance in the risk networks of people who inject drugs
Source: PLoS One. 2020 Jun 22;15(6):e0235124. doi: 10.1371/journal.pone.0235124 (PMC7307734; doi:10.1371/journal.pone.0235124)
Supplement: S1 Appendix — (PDF) [file pone.0235124.s001.pdf]

S1 Appendix. Composition of the needle sharing network

As described in the text, the network we analyze here is composed of 117 PWID who participated in both the Phase 2 questionnaire and network supplement interview. Here we provide some additional details of the sample and how we generated the network.

Of the 117 individuals in the final network, 33 were key respondents. The 33 key respondents named 96 individuals we were able to uniquely identify, 12 of whom were other key respondents. Of the remaining 84 named partners of the key respondents, we interviewed 69 and could not or did not contact the remaining 15 (many of whom were unavailable for reasons such as incarceration or treatment). An additional 15 participants were brought to the study by key respondents. Individuals who were named by key respondents but could not be contacted for an interview are excluded from the network, as are individuals named by the second wave of participants (i.e., non key-respondents) who had not already been named by key respondents.

The needle-sharing network is a subset of the injection drug co-use network. Our survey instrument only allowed for respondents to name up to nine co-injection partners, however no respondents who named nine partners had needle-sharing ties with all of their reported co-injection partners. Consequently, we believe that the cap on number of co-injection partners respondents were allowed to name had a limited impact, if any, on the degree distribution of the needle-sharing network. Reduction of the network to only Phase 2 participants (instead of including all individuals who were named, even if they did not participate in the study) reduced the in-degree of study participants by no more than two ties and by a mean of 0.11 ties. Roughly half of people in the network (n=61/117) did not have any receptive needle sharing ties; amongst these, many (n=47) were complete isolates, meaning they did not (and were not reported by others) as sharing a needle with anyone else in the Phase 2 sample, as either the first or second user. Only one individual in the network who had a receptive needle-sharing tie in the complete sample became an in-degree isolate when we reduced the

network to Phase 2 participants only.

We take the union of all reported ties as the edgeset for the network. We note, however, that the concordance of reports of needle-sharing by participants in the study is very low. Only 15 of 151 ties (10%) were reported by both respondents in a dyad. In contrast, concordance of reports of co-injection partnerships, i.e., using drugs together but not necessarily sharing equipment, is 46%. Some of this low concordance can likely be attributed to lack of perfect overlap in the time frames of sampling for co-injection ties. However, we suspect that some portion of this mismatch between respondents reflects a bias due to the social (in)desirability of needle-sharing; with some individuals being more willing to report this activity than others. Respondents more often reported that their co-injection partners used needles after them (79/151 ties) than that they themselves had used a needle after someone else (57/151 ties), a pattern which may also reflect a social desirability bias. This issue should be addressed in future research but is partially mitigated by our double-sampling approach (asking respondents both who they used after and who used a needle after them), and is why we take all reported ties rather than only concordant ties as the basis for analysis.
