# Supplementary material for: Competing forces of withdrawal and disease avoidance in the risk networks of people who inject drugs
Source: PLoS One. 2020 Jun 22;15(6):e0235124. doi: 10.1371/journal.pone.0235124 (PMC7307734; doi:10.1371/journal.pone.0235124)
Supplement: S2 Appendix — (PDF) [file pone.0235124.s002.pdf]

S2 Appendix. HIV and HCV data

The HIV and HCV data come from respondent self-reports of previous HIV and HCV tests (and treatment, if applicable). Many respondents had previously participated in rapid HIV and HCV testing with our team as part of Phase 1 of the study, and we conducted rapid testing again in Phase 2. We use self-reports as the basis for analysis, as we expect respondents' beliefs about their status to condition their behavior, even though there may be mismatches between their beliefs and actual infection status. Here we summarize discrepancies between the results of Phase 2 HIV and HCV rapid testing and the self-report data (Table A1).

As described in the text, four percent of respondents in the network (5/117) tested HIV positive in our rapid testing, and all participants we tested had accurate knowledge of their HIV status. Only one individual in the Phase 2 sample had never been previously tested for HIV. For HCV, 68% of respondents (80/117) reported that they had previously tested positive for HCV (without reporting having been cured), and four individuals reported having been cured of a previous HCV infection. Six individuals (5%) reported that they had never been tested for HCV. However, 85% of participants in Phase 2 HCV testing (93/110) tested positive for HCV antibodies (see Table A1).

We can summarize the differences between our test results and self-reported HCV status as follows: (1) one individual tested negative but stated that he knew he was HCV-positive but did not have antibodies; (2) four individuals tested positive for antibodies but stated that they had been previously treated for HCV and told by a doctor that they had been cured; and (3) 12 participants reported that they has never tested positive for HCV but subsequently tested positive in our rapid tests. Of these 12, one individual had previously tested negative in Phase 1 and so represents a probable new infection. Two were new participants in Phase 2, and so we have no independent information on their previous tests. But nine participants reported that they had never tested positive for HCV even though they had tested positive and been informed of their positive test during Phase 1 of our study. Five of these individuals

HIV

	Test result	
**Self-report**	−	+
−	106	0
+	0	5
Unknown	1	0

HCV

	Test result	
**Self-report**	−	+
−	16	12
+	0	76
Unknown	1	5

Table A1: Cross-tabulations of participants' self-reports of their HIV and HCV status with results of rapid antibody tests. Five respondents did not participate in HIV testing, and six respondents did not participate in HCV testing. They are excluded from these tables. Note that the "Neg/Neg" category for HCV includes four individuals who reported that they had been cured of a past infection, and the "Pos/Pos" category includes one individual who reported an HCV infection but did not test positive for antibodies.

reported taking an additional test in the intervening period between their Phase 1 and Phase 2 tests. This may mean that they confirmed that they did not have an active infection, although it does not explain why they claimed to never have tested positive in the past. It is probable that some of the positive results are from individuals who spontaneously cleared HCV infections in the past (and so show antibodies despite not being infected), but we do not have sufficient information to determine if this could be the case.

To generate Figure 3, we determined the "test" status of the seven individuals who did not participate in Phase 2 testing for HCV using our testing results from Phase 1 (for six of seven participants), and a self-reported recent test result for one individual.
