# Supplementary material for: Competing forces of withdrawal and disease avoidance in the risk networks of people who inject drugs
Source: PLoS One. 2020 Jun 22;15(6):e0235124. doi: 10.1371/journal.pone.0235124 (PMC7307734; doi:10.1371/journal.pone.0235124)
Supplement: S3 Appendix — (PDF) [file pone.0235124.s003.pdf]

S3 Appendix. Additional ERGM summary tables

	Partner Restriction			Informed altruism		
	Coef.	S.E.	p-value	Coef.	S.E.	p-value
Edges	−6.44	0.63	<0.01	−6.40	0.51	<0.01
Node in-factor Female	0.23	0.34	0.49	0.23	0.34	0.49
Nodematch Female	0.67	0.36	0.07	0.67	0.36	0.07
Node in-factor Age	−0.01	0.01	0.41	−0.01	0.01	0.36
Absdiff Age	−0.01	0.01	0.06	−0.01	0.01	0.06
Node in-factor Location 4	−1.80	0.52	<0.01	−1.81	0.53	<0.01
Nodematch Location 4	2.80	0.52	<0.01	2.80	0.52	<0.01
Node in-factor Injection 1–3×	0.65	0.35	0.06	0.64	0.35	0.07
Node in-factor Injection 4–7×	1.09	0.35	<0.01	1.09	0.35	<0.01
Node in-factor Injection 8+×	1.36	0.38	<0.01	1.36	0.38	<0.01
Mutual	6.45	0.37	<0.01	6.45	0.37	<0.01
GWESP ($\alpha = 0.04$)	0.58	0.12	<0.01	0.58	0.12	<0.01
Node in-factor HIV−	0.01	0.29	0.96			
Node out-factor HIV+				0.07	0.32	0.82
AIC	950.73			950.63		
BIC	1048.43			1048.34		
Log Likelihood	−462.36			−462.32		

Table A1: ERGM results for HIV models.

	Partner Restriction			Informed Altruism		
	Coef.	S.E.	p-value	Coef.	S.E.	p-value
Edges	−6.36	0.55	<0.01	−6.39	0.55	<0.01
Node in-factor Female	0.26	0.36	0.47	0.24	0.36	0.50
Nodematch Female	0.66	0.40	0.10	0.64	0.40	0.11
Node in-factor Age	−0.01	0.01	0.32	−0.00	0.01	0.69
Absdiff Age	−0.01	0.01	0.11	−0.02	0.01	0.05
Node in-factor Location 4	−1.78	0.57	<0.01	−1.83	0.57	<0.01
Nodematch Location 4	2.84	0.56	<0.01	2.83	0.57	<0.01
Node in-factor Injection 1–3×	0.67	0.37	0.07	0.59	0.38	0.12
Node in-factor Injection 4–7×	1.13	0.37	<0.01	1.08	0.38	<0.01
Node in-factor Injection 8+×	1.40	0.40	<0.01	1.35	0.41	<0.01
Mutual	6.44	0.38	<0.01	6.45	0.38	<0.01
GWESP ($\alpha = 0.04$)	0.56	0.13	<0.01	0.56	0.13	<0.01
Node in-factor HCV−	−0.16	0.17	0.37			
Node out-factor HCV+				−0.19	0.16	0.25
AIC	949.84			949.34		
BIC	1047.54			1047.04		
Log Likelihood	−461.92			−461.67		

Table A2: ERGM results for HCV models.
